# Serum microRNA Levels as a Biomarker for Diagnosing Non-Alcoholic Fatty Liver Disease in Chinese Colorectal Polyp Patients

**DOI:** 10.3390/ijms24109084

**Published:** 2023-05-22

**Authors:** Lui Ng, Ryan Wai-Yan Sin, David Him Cheung, Wai-Keung Leung, Abraham Tak-Ka Man, Oswens Siu-Hung Lo, Wai-Lun Law, Dominic Chi-Chung Foo

**Affiliations:** 1 Department of Surgery, School of Clinical Medicine, Li Ka Shing Faculty of Medicine, The University of Hong Kong, Hong Kong SAR, China; ryansin@connect.hku.hk (R.W.-Y.S.); cheung96@hku.hk (D.H.C.); tkman@hku.hk (A.T.-K.M.); oswenslo@yahoo.com (O.S.-H.L.); lawwl@hku.hk (W.-L.L.); 2 Department of Medicine, School of Clinical Medicine, Li Ka Shing Faculty of Medicine, The University of Hong Kong, Hong Kong SAR, China; waikleung@hku.hk

**Keywords:** NAFLD, colorectal polyp, serum microRNA (miRNA), diagnostic biomarker

## Abstract

Non-alcoholic fatty liver disease (NAFLD) is one of the most common chronic liver diseases and its prevalence is increasing worldwide. It is reported that NAFLD is associated with colorectal polyps. Since identifying NAFLD in its early stages could prevent possible disease progression to cirrhosis and decrease the risk of HCC by early intervention, patients with colorectal polyp may thus be considered a target group for screening NAFLD. This study aimed to investigate the potential of serum microRNAs (miRNAs) in identifying NAFLD for colorectal polyp patients. Serum samples were collected from 141 colorectal polyp patients, of which 38 had NAFLD. The serum level of eight miRNAs was determined by quantitative PCR and delta Ct values of different miRNA pairs which were compared between NAFLD and control groups. A miRNA panel was formulated from candidate miRNA pairs by multiple linear regression model and ROC analysis was performed to evaluate its diagnostic potential for NAFLD. Compared to the control group, the NAFLD group showed significantly lower delta Ct values of miR-18a/miR-16 (6.141 vs. 7.374, *p* = 0.009), miR-25-3p/miR-16 (2.311 vs. 2.978, *p* = 0.003), miR-18a/miR-21-5p (4.367 vs. 5.081, *p* = 0.021) and miR-18a/miR-92a-3p (8.807 vs. 9.582, *p* = 0.020). A serum miRNA panel composed of these four miRNA pairs significantly identified NAFLD in colorectal polyp patients with an AUC value of 0.6584 (*p* = 0.004). The performance of the miRNA panel was further improved to an AUC value of 0.8337 (*p* < 0.0001) when polyp patients with other concurrent metabolic disorders were removed from the analysis. The serum miRNA panel is a potential diagnostic biomarker for screening NAFLD in colorectal polyp patients. This serum miRNA test could be performed for colorectal polyp patients for early diagnosis and for prevention of the disease from progressing into more advanced stages.

## 1. Introduction

Non-alcoholic fatty liver disease (NAFLD) is one of the most common chronic liver diseases and its prevalence is increasing worldwide, being estimated to affect up to 25% of the global population [1]. Comparing to other metabolic disorders such as obesity and diabetes which have multiple well-known risk factors as predictors, a substantial portion of individuals with normal body mass index (BMI) have NAFLD [2]. Initiating from simple hepatic steatosis (NAFL, nonalcoholic fatty liver), and although remission can easily be achieved, NAFLD is difficult to diagnose due to a lack of characteristic symptoms [3]. Approximately 30% to 40% of NAFLD patients develop nonalcoholic steatohepatitis (NASH), which is characterized by excessive accumulation of fat coupled with the development of inflammation. Without proper treatment, patients are subjected to the risk of the disease developing into fibrosis, cirrhosis or hepatocellular carcinoma over time [4]. It is suggested that NAFLD or more specifically NASH might have already become the most common cause of liver diseases and liver-related deaths globally [5].

Colorectal polyp is a benign growth on the mucosal surface of the colon or rectum, and over a period of 5 to 15 years, can develop into colorectal cancer (CRC) [6], which is one of the most common cancers and a leading cause of cancer-related death worldwide [7]. Hence, screening of polyps is crucial for reducing the morbidity of CRC. Adenomatous polyps are considered the precursor lesions for the majority of CRCs through an adenoma-carcinoma sequence, whereas recent studies also proposed that hyperplastic polyps contribute to CRCs through serrated or microsatellite instable pathways [8]. The prevalence of colorectal polyp is rapidly increasing worldwide in recent years, which may be influenced by factors such as changes in diet and lifestyle habits [9]. A US study reported that the prevalence of colorectal polyp varies in different geographical locations and races, with the highest incidence in Western countries and the lowest in Africa and South-Central Asia [10]. Recently, more Asian studies investigating prevalence and risk factors for colorectal polyps and CRC have been published. The prevalence of colorectal polyps in an average risk population for Asian countries is around 20% and generally with higher prevalence in older age and male gender [8,11,12]. Recent studies have reported some risk factors for adenomatous polyps, including age, gender, metabolic syndrome, Helicobacter pylori infection, smoking, alcohol drinking, etc. [13,14,15].

Researches have been investigating whether NAFLD has a relationship to colorectal polyps, since metabolic syndrome and insulin resistance are associated with a higher risk of colon cancer, whereas NAFLD is regarded as a manifestation of metabolic syndrome in the liver [16]. In line with this hypothesis, epidemiological research reported that the occurrence of NAFLD is associated with colorectal polyps [16,17,18]. Though the underlying mechanism of the association between NAFLD and colorectal polyps is still unclear, the significant roles of insulin resistance, inflammatory response and obesity in the pathophysiological pathways of NAFLD and colorectal polyps have been suggested [19]. The levels of hormones are an important factor for the development of colorectal neoplasms in patients with NAFLD. For example, insulin resistance, which is a risk factor for NAFLD, is closely related to colorectal polyp and colorectal cancer [20]. Moreover, high serum levels of pro-inflammatory cytokines, such as those including tumor necrosis factor (TNF)-α, interleukin (IL)-6 and IL-8, are associated with both NAFLD, and colorectal neoplasm patients with NAFLD are significantly higher than those in the normal population, which have been found to be significantly associated with the risk of developing colorectal neoplasms [21]. Similarly, the high prevalence of a high-energy diet and obesity has played a role in the increase in the prevalence of both NAFLD and colorectal neoplasms [22]. On the other hand, serum albumin is a recognized biomarker for assessing the nutritional condition. Chronological decline in serum albumin concentration was found to be indicator of the occurrence of serious complications of NAFLD/NASH and development of colorectal polyps [8,23], yet the causality requires further investigations. The prevalence of NAFLD was higher in the colorectal polyp group when compared to the control group [16], and an increased risk for NAFLD was more evident in patients with a greater number of adenomatous polyps [16]. Since identifying NAFLD in its early stages could prevent possible disease progression to cirrhosis and decrease the risk of HCC by early intervention, patients with colorectal polyp may thus be considered a high-risk group, and screening of NAFLD will be highly beneficial.

The gold standard for diagnosing and monitoring NAFLD is liver biopsy [24], yet it is not feasible to all colorectal polyp patients within clinical practice due to its cost and invasiveness [25]. In addition, there remains a risk of serious complications despite liver biopsy being considered safe, including pain at the biopsy site, serious bleeding and, rarely, death [26]. Therefore, a practical screening biomarker is warranted for screening colorectal polyp patients with a high risk of NAFLD.

Circulating blood microRNAs (miRNAs) are endogenous, noncoding, small RNAs present in blood. Due to their size, abundance, tissue specificity, relative stability in peripheral circulation and dysregulated levels associated with different diseases, they offer great potential as noninvasive screening biomarkers [27]. There have been several studies demonstrating the clinical significance of serum miRNA in monitoring NAFLD progression. For instance, serum levels of certain miRNAs showed high accuracy for distinguishing NASH from simple steatosis or have the potential for steatohepatitis diagnosis [28,29,30]. Furthermore, serum levels of certain hepatic miRNAs were able to predict the percentage of liver fat with errors of less than 5% [31]. However, no studies have identified serum miRNAs for screening NAFLD specifically in colorectal polyp patients which are considered the high-risk group for the disease. Hence, this study aimed to identify serum miRNAs which show differential levels between colorectal polyp patients with or without NAFLD and evaluate the potential of serum miRNAs in identifying NAFLD in patients with colorectal polyps.

## 2. Results

### 2.1. Patient Characteristics

A total of 141 Chinese colorectal polyp patients, confirmed by colonoscopy, were included in this study, including 38 patients diagnosed with NAFLD as the NAFLD group, and 103 patients confirmed with absence of fatty liver or other liver diseases as the control group. As shown in Table 1, among the 38 NAFLD patients, 57.9% were male and the mean age of these patients was 62.2 ± 8.50 years. Among the 103 control subjects, 53.8% were male and the mean age of these patients was 64.9 ± 9.96 years. Regarding the type of polyps, tubular adenoma was the most prevalent in both groups, followed by hyperplastic polyp. The presence of other concurrent metabolic disorders including diabetes and obesity/overweight (BMI ≥ 30) were obtained from the patients. Within the NAFLD patient group, 8 of them (20.0%) were diagnosed with diabetes, 5 (13.2%) with overweight/obesity and 11 (28.9%) with both diseases. Within the control group, 13 of them (12.6%) had diabetes, 14 (13.5%) with obesity/overweight and 9 (8.7%) had both diseases.

### 2.2. Comparison of Different miRNA Pairs between Polyp Patients with or without NAFLD

**Figure 1:**
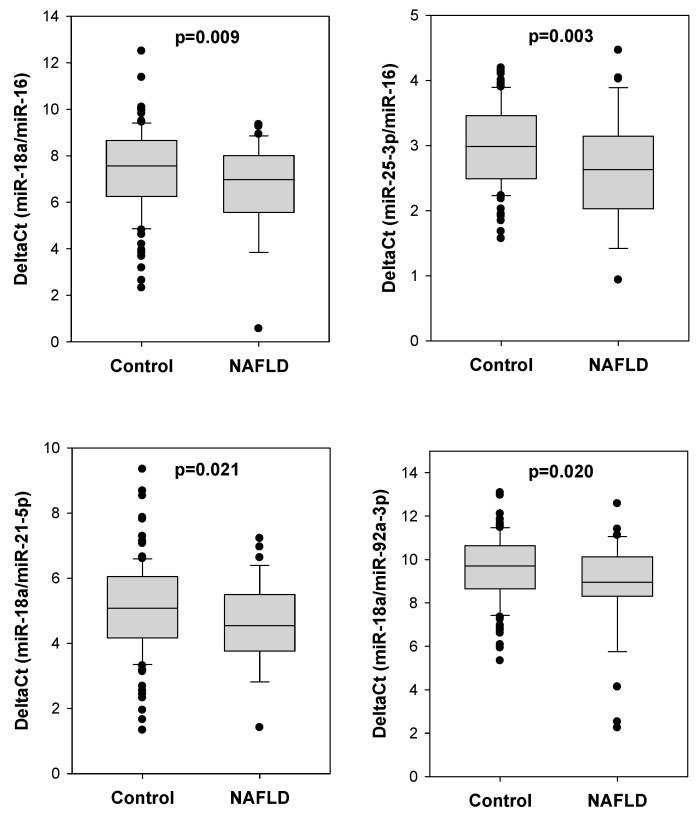
Four serum miRNA pairs showing differential delta Ct values between NAFLD and control groups. The delta Ct values of four serum miRNA pairs, including miR-18a/miR-16, miR-25-3p/miR-16, miR-18a/miR-21-5p and miR-18-3p/miR-92a-3p, were significantly lower in colorectal polyp patients with NAFLD (N = 38) when compared to NAFLD-free patients (control; N = 103).

We determined the serum levels of eight miRNAs including miR-16, miR-18a-5b, miR-21-5p, miR-25-3p, miR-92a-3p, miR-93b-5p, miR-106-5p and miR-1246. We found that the delta Ct values of certain miRNA pairs were significantly lower in colorectal polyp patients with NAFLD (Figure 1). Compared to colorectal polyp patients without NAFLD, those with NAFLD showed significantly lower delta Ct values of miR-18a/miR-16 (6.141 vs. 7.374, *p* = 0.009), miR-25-3p/miR-16 (2.311 vs. 2.978, *p* = 0.003), miR-18a/miR-21-5p (4.367 vs. 5.081, *p* = 0.021) and miR-18a/miR-92a-3p (8.807 vs. 9.582, *p* = 0.020).

**Figure 2:**
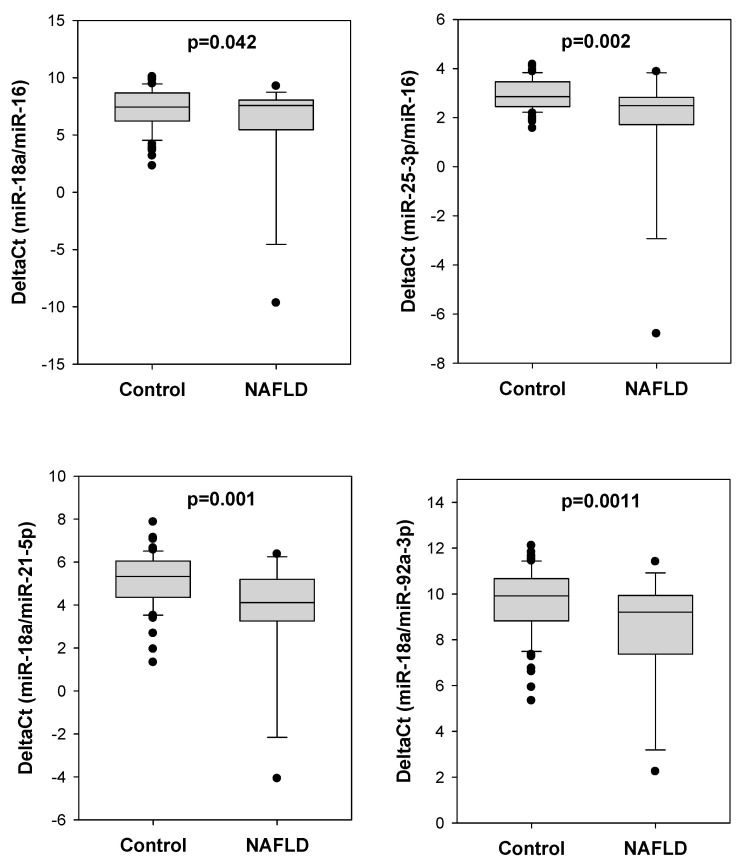
Delta Ct values of the four differential serum miRNA pairs in NAFLD and control groups without concurrent diabetes nor obesity/overweight. The four serum miRNA pairs—miR-18a/miR-16, miR-25-3p/miR-16, miR-18a/miR-21-5p and miR-18a/miR-92a-3p—generally showed a larger difference in delta Ct values between the NAFLD group (N = 14) and control group (N = 67) after colorectal polyp patients with other concurrent metabolic disorders (diabetes and obesity/overweight) were removed from the analysis.

Moreover, in addition to NAFLD, 60 polyp patients (24 from NAFLD group; 36 from control group) were diagnosed with other concurrent metabolic disorders including diabetes and obesity or overweight. To rule out the potential interference of concurrent metabolic diseases on serum miRNA levels [32], we refined polyp patients to the NAFLD group and control group without other concurrent metabolic diseases. Using such approach, there was generally a larger difference in delta Ct value of different miRNA pairs between the NAFLD group and control group (Figure 2): miR-18a/miR-16 (5.708 vs. 7.287, *p* = 0.042), miR-25-3p/miR-16 (1.820 vs. 2.940, *p* = 0.002), miR-18a/miR-21-5p (3.579 vs. 5.135, *p* = 0.001) and miR-18a/miR-92a-3p (8.378 vs. 9.678, *p* = 0.011).

### 2.3. Diagnostic Performance of Serum miRNA Panel for NAFLD in Colorectal Polyp Patients

**Figure 3:**
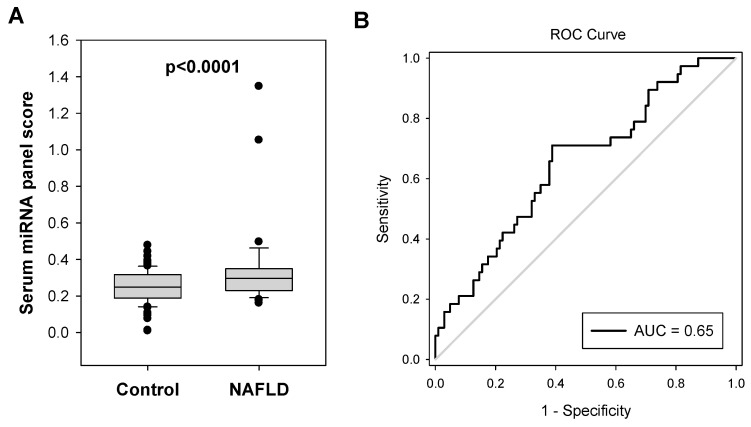
Serum miRNA panel as a potential biomarker for screening NAFLD in colorectal polyp patients. (**A**) A serum miRNA panel which was formulated from the 4 serum miRNA pairs using multiple linear regression 1.088 + (0.0302 × miR-18a/miR-16) − (0.118 × miR-25-3p/miR-16) + (0.00378 × miR-18a/miR-21-5p) − (0.0714 × miR-18a/miR-92a-3p) showed significantly higher value (*p* < 0.0001) in NAFLD group (N = 38) compared to the control group (N = 103). (**B**) ROC analysis showed that the serum miRNA panel significantly identify NAFLD in colorectal polyp patients with an AUC value of 0.66.

We applied multiple linear regression analysis to formulate a combination of these four miRNA pairs for diagnosing NAFLD in colorectal polyp patients: serum miRNA panel score = 1.088 + (0.0302 × miR-18a/miR-16) − (0.118 × miR-25-3p/miR-16) + (0.00378 × miR-18a/miR-21-5p) − (0.0714 × miR-18a/miR-92a-3p). The miRNA panel score in the NAFLD group was significantly higher than that of the control group (0.365 vs. 0.268, *p* < 0.0001, Figure 3A). As shown in Figure 3B, the serum miRNA panel significantly identified NAFLD in colorectal polyp patients with an AUC value of 0.6584 (95% CI, 0.5578 To 0.7590; *p* = 0.0040).

**Figure 4:**
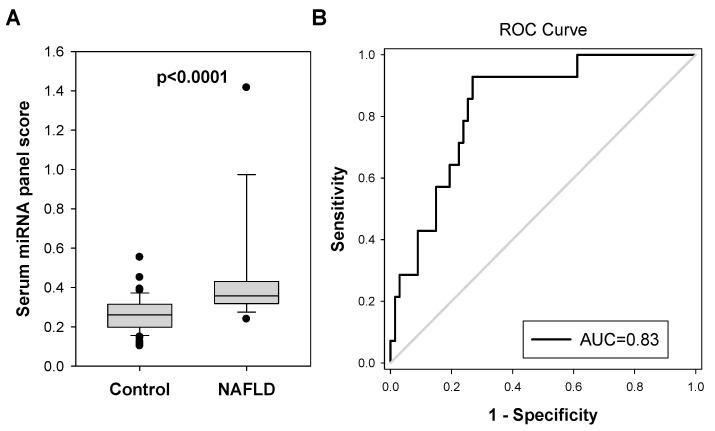
Serum miRNA panel showed better performance in identifying NAFLD in colorectal polyp patients without concurrent diabetes nor obesity/overweight. (**A**) The serum miRNA panel showed an improved significant difference (*p* < 0.0001) between the NAFLD group (N = 14) and control group after colorectal polyp patients (N = 67) with other concurrent metabolic disorders (diabetes and obesity/overweight) were removed from the analysis. (**B**) ROC analysis showed that the AUC of such miRNA panel for NAFLD diagnosis was increased to 0.8337 after colorectal polyp patients with concurrent metabolic disorders (diabetes and obesity/overweight) were removed from the analysis.

Furthermore, when colorectal polyp patients with other concurrent metabolic diseases were removed, this further extended the significant difference between miRNA panel scores in NAFLD and control patients (0.438 vs. 0.263, *p* < 0.0001, Figure 4A). The AUC of this miRNA panel for NAFLD diagnosis was increased to 0.8337 (95% CI, 0.7329 To 0.9345; *p* < 0.0001, Figure 4B). The best combination of sensitivity and specificity which was at the cut-off value of 0.3066 was 92.9% and 73.1%, respectively.

### 2.4. Validation of Serum miRNA Panel for Diagnosing Probable NAFLD in Another Small Cohort of Colorectal Polyp Patients

**Figure 5:**
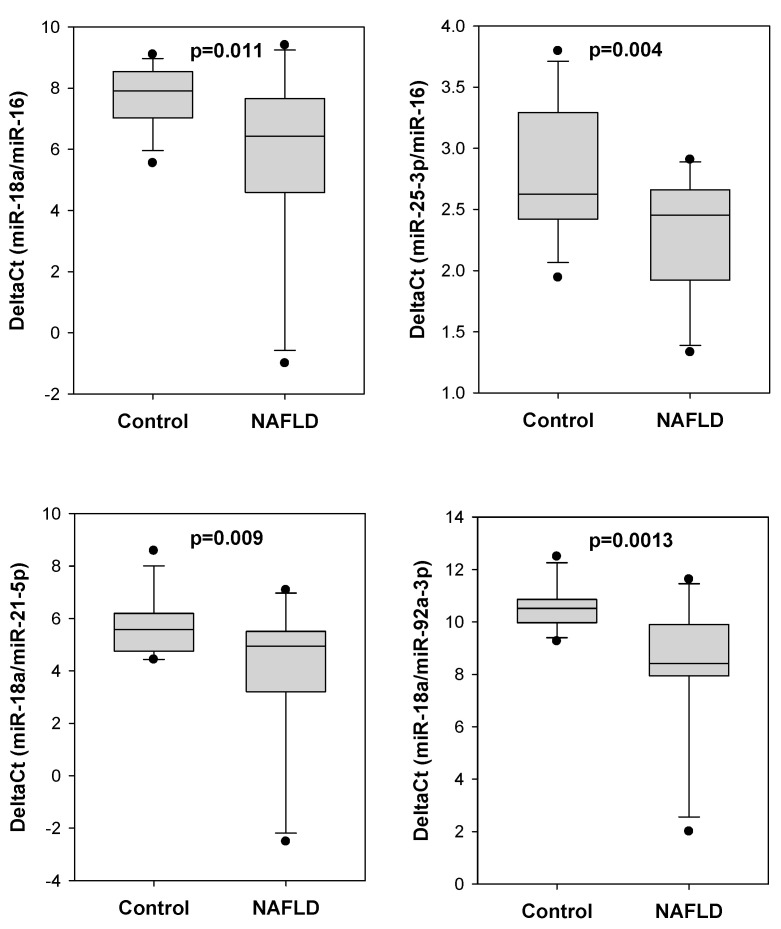
Four serum miRNA pairs showing differential delta Ct values between NAFLD and control groups in an independent cohort of colorectal polyp patients. The delta Ct values of four serum miRNA pairs, including miR-18a/miR-16, miR-25-3p/miR-16, miR-18a/miR-21-5p and miR-18a/miR-92a-3p, were significantly lower in colorectal polyp patients with NAFLD (N = 10) when compared to those without NAFLD (control; N = 12).

Finally, we examined the performance of the serum miRNA panel in a validation cohort consisting of 22 Chinese colorectal polyp patients who had been scanned for fatty liver by ultrasound. Twelve of them showed no sign of fatty liver, whereas 10 of them were probable NAFLD patients with fatty liver or liver cyst. The characteristics of these patients are shown in Table 2. We determined the values of miRNA pairs for these samples. In line with the above findings, probable NAFLD patients showed significantly lower delta Ct values of miR-18a/miR-16 (Figure 5, 5.823 vs. 7.758, *p* = 0.043), miR-25-3p/miR-16 (2.304 vs. 2.785, *p* = 0.042), miR-18a/miR-21-5p (4.035 vs. 5.719, *p* = 0.046) and miR-18a/miR-92a-3p (8.288 vs. 10.592, *p* = 0.007).

**Figure 6:**
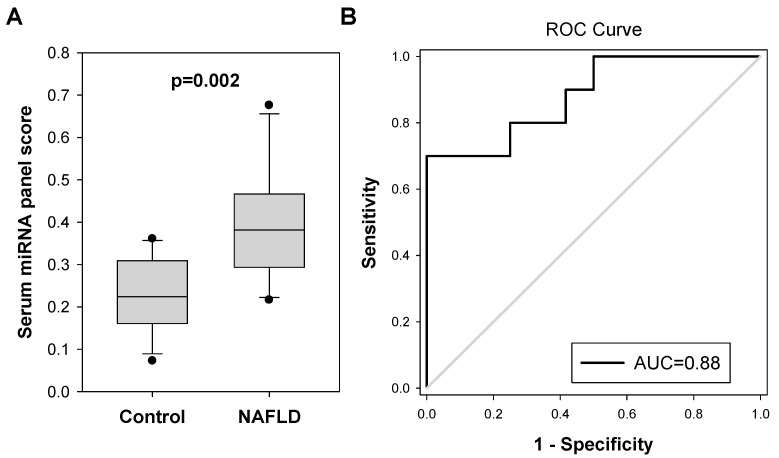
Serum miRNA panel showed consistent performance in identifying probable NAFLD in an independent cohort of colorectal polyp patients. (**A**) The serum miRNA panel score was significantly higher in the probable NAFLD group (median = 0.393; N = 10) when compared to the control group (median = 0.230, *p* = 0.002; N = 12). (**B**) ROC analysis showed that the AUC of the serum miRNA panel for NAFLD diagnosis was 0.8800.

The serum miRNA panel score for these patients was determined. As shown In Figure 6, the score was significantly higher in the probable NAFLD group (0.393) when compared to the control group (0.230, *p* = 0.002). We examined the performance of the serum miRNA test in identifying polyp patients with probable NAFLD disease based on the cut-off value of 0.3066. Eight out of ten patients were correctly identified with probable NAFLD (sensitivity = 80.0%), whereas nine out of twelve normal subjects were correctly identified as normal (specificity = 75.0%). These findings confirmed the serum miRNA panel showing consistent performance in diagnosing NAFLD patients.

## 3. Discussion

NAFLD is significantly associated with the presence of colorectal polyps. Therefore, it is suggested that polyp patients should be screened for the presence of NAFLD to prevent disease progression to cirrhosis and decrease the risk of HCC by early intervention. One major advantage of our serum miRNA approach as a screening biomarker is that serum samples are routinely collected from colorectal polyp patients when they have a follow-up at hospitals. Our results showed that the delta Ct values of four miRNA pairs in serum samples, namely miR-18a/miR-16, miR-25-3p/miR-16, miR-18a/miR-21-5p and miR-18a/miR-92a-3p, were significantly lower in colorectal polyp patients with NAFLD when compared to those without NAFLD. A miRNA panel formulated from these four miRNA pairs was able to identify NAFLD in colorectal polyp patients with an AUC value of 0.6584. These findings suggested that our serum miRNA panel is a potential biomarker for screening NAFLD in colorectal polyp patients. More importantly, when patients with other metabolic disorders (i.e., diabetes and obesity or overweightness) were removed from the analysis, the AUC value was further increased to 0.8337. This finding suggests that colorectal polyp patients who measured with high serum miRNA panel scores (i.e., above 0.3066 which showed the best combination of sensitivity and specificity in our patient cohort), in particular for those without diabetes nor obesity/overweight, can be classified as the high-risk group, and further verification of the occurrence of NAFLD is recommended. We further validated these findings using an independent cohort of colorectal polyps patients who potentially develop a fatty liver or cyst, but have not tested for other metabolic diseases. In line with the aforementioned findings, our data suggested that the delta Ct level of the four miRNA pairs was lower in probable NAFLD patients when compared to control patients. More importantly, the serum miRNA panel score was able to identify probable NAFLD patients with 80.0% sensitivity and 75% specificity, indicating the serum miRNA panel exhibits consistent performance in diagnosing NAFLD patients.

In this study, we evaluated the potential of applying serum miRNA levels for identifying NAFLD in colorectal polyp patients. Due to the low level of circulating miRNAs in the peripheral blood, effective data normalization using RT-qPCR is somewhat challenging. We tested different normalization strategies including endogenous controls that display stable expression across all samples (e.g., U6, miR-16, miR-39) [33] or spike-in cel-miR-39. Though the normalized levels of certain miRNAs showed statistically significant differences between NAFLD and normal groups, the variation was unfavorably high among samples within the same group, which greatly affected the performance of the serum miRNA test. Alternatively, we found that the delta Ct values of certain miRNA pairs were significantly distinct between NAFLD and control groups, and they showed more consistent levels among study subjects within each group. A similar strategy has been adopted in several blood biomarker studies for different diseases or cancers [33,34,35,36,37,38]. Moreover, this miRNA-pair-based normalization method presents a better ability to identify more biomarkers as candidates, in comparison to those using internal controls or spike-in as the normalizing agent [33]. In line with their findings, our data showed that for individual miRNA normalized by blood volume, only miR-16 and miR-21 showed significant difference between NAFLD and control groups (Supplementary File S1), whereas for miRNA-pair approach, there were four miRNA-pairs showing significant difference, and the *p*-value was also more significant (*p* = 0.003 to 0.021) than that of individual miR-16 or miR-21 normalized by blood volume (*p* = 0.040 and 0.045, respectively). Hence, our study provides further support to the use of miRNA pair in biomarker identification study, and hopefully more potential biomarkers can be identified with this approach.

Our serum miRNA panel is composed of miR-16, miR-18a, miR-21-5p, miR-25-3p and miR-92a-3p. These noncoding RNAs have been reported to be associated with NAFLD or liver disease. MiR-16 and miR-21 are commonly deregulated in liver fibrosis and hepatocellular carcinoma [39]. Serum miR-16 level was elevated in NAFLD patients with simple steatosis (NAFLD-SS) when compared to the control group [39] and its upregulated level was associated with NAFLD/NASH progression [40], but another study indicated that the serum level of miR-16-5p was lower in the group of patients with steatosis [29]. MiR-18a was significantly downregulated in the liver of mice with NAFLD [41]. Serum miR-21 levels in NAFLD patients, when compared to controls, varied depending on the studies [42]. One study suggested that the serum miR-21 level was lower in 25 NAFLD patients than the expressions in 12 healthy controls [43], whereas another study claimed that the serum level of miR-21 was higher in patients with NAFLD [44]. Serum miR-25-3p expression is significantly suppressed in NASH patients with significant fibrosis compared to NAFLD patients [45], indicating its association with NAFLD progression. Moreover, a decrease in serum miR-25-3p level was also associated with poor survival in patients with liver disease [46]. MiR-92a-3p was downregulated in the liver tissues in aged NAFLD rat model [47], and serum level of such miRNA was associated with different stages of liver steatosis [48].

One main limitation of this study is the relatively small sample size. Due to logistic issues, serum samples were collected from all patients prior to the colonoscopy examination. After confirmation of their colonoscopy result and diagnostic result for the presence/absence of NAFLD or other metabolic diseases including diabetes and obesity/overweight, the refined number of the NAFLD group and control group was 38 and 103, respectively. To validate our finding, we retrieved an independent cohort of 22 colorectal polyp patients whose serum samples were collected in 2013. Among them, 12 were confirmed to be free of fatty liver disease, whereas 10 of them were probable NAFLD patients who carried fatty liver disease or liver cyst. Though the sample size of this independent cohort was small, we were able to validate our findings. The four serum miRNA pairs showed a significant difference between probable NAFLD group and the control group, and the values for both groups were comparable to that obtained in the previous cohort. Moreover, the serum miRNA panel was able to effectively identify colorectal polyp patients with probable NAFLD in this validation cohort with the sensitivity of 80% and specificity of 75.0%. Another limitation of this study is that all NAFLD patients recruited were at stage 1 (simple fatty liver or steatosis). Although it is an advantage for the serum miRNA panel to detect patients with NAFLD at early stage, it is warranted to investigate whether higher stage disease would affect the performance of serum miRNA panel. The last limitation of this study is that certain variables that might affect the profile of serum miRNA were not investigated and might affect the accuracy of the test in some patients. As we demonstrated in our study, the precision of the serum miRNA test for identifying NAFLD in colorectal polyp patients was reduced when the patients had other concurrent metabolic diseases including diabetes and obesity/overweight. This is mainly caused by the serum miRNA profiles being affected by these metabolic disorders [49]. However, NAFLD is a multisystem disease and is closely associated with many complications which are not investigated in this study, including viral infections (HCV, HIV) [50,51], bacterial infection [52,53], and diseases in various systems such as cardiovascular disease, chronic kidney disease, psoriasis, polycystic ovarian syndrome, chronic intermittent hypoxia of obstructive sleep apnea syndrome, and hypothyroidism [52,54].

To summarize, this study demonstrated that our serum miRNA panel, which is made up of miR-18a/miR-16, miR-25-3p/miR-16, miR-18a/miR-21-5p and miR-18a/miR-92a-3p, is a potential non-invasive biomarker for identifying NAFLD in colorectal polyp patients. Since patients with colorectal polyp have a higher risk of concurrent NAFLD, we suggest serum miRNA test could be performed for these patients for early diagnosis and to prevent the disease from progressing to cirrhosis by early intervention, hence reducing the risk of HCC. To improve the clinical value of this serum miRNA panel, in the future we plan to recruit a larger cohort of colorectal polyp patients with different stages of NAFLD for constructing a more comprehensive nomogram by including other variables including complete blood count, liver enzyme level, the complication of other infections and diseases to improve the performance of the serum miRNA panel in screening the presence of NAFLD in colorectal polyp patients.

## 4. Materials and Methods

### 4.1. Patient Specimens

This was a case-controlled study conducted in the departments of Surgery and Medicine of Queen Mary Hospital of Hong Kong. Serum samples were collected from all patients prior to the colonoscopy examination after obtaining written informed consent for participation in accordance with the Declaration of Helsinki through protocols approved by the Institutional Review Board of the University of Hong Kong/Hospital Authority Hong Kong West Cluster. One hundred forty-one colorectal polyp patients who were diagnosed with colorectal polyp by colonoscopy examination were recruited into this study. The presence of NAFLD was diagnosed by blood liver function test and ultrasound scan. The liver was scanned by ultrasound diagnostic instrument and conducted by an experienced sonographer who was blinded to the clinical details of the patients. Radiologists who performed the ultrasound evaluation were blinded to the laboratory data. According to 2010 guidelines for the diagnosis of NAFLD, patients with viral hepatitis, autoimmune hepatitis, drug-induced hepatitis, various liver cirrhosis and alcoholic liver diseases were excluded. The diagnosis of NAFLD was established if the ultrasonogram showed increased echogenicity when compared to the renal parenchyma [55]. Thirty-eight colorectal polyp patients diagnosed with NAFLD (simple fatty liver or steatosis) were sorted into the NAFLD group, whereas 103 colorectal polyp patients free of NAFLD based on their blood liver function test and ultrasound scan were sorted into the control group (Supplementary File S1). Other metabolic disorders including diabetes and obesity/overweight were retrieved from hospital’s clinical database. Three millilitres of blood was taken by Serum Vacuum Blood Collection Tubes, followed by the extraction of serum. Blood tubes were first centrifuged at 3000 rpm for 10 min at 4 °C, and the upper layer of serum was collected. Further centrifugation at 10,000× *g* for 10 min at 4 °C was performed to remove any remaining clot before serum was transferred to a sterile polypropylene tube and was stored at −80 °C until use.

### 4.2. Serum miRNA Extraction

The serum miRNA was extracted using a combination of TRIzol™ LS Reagent (Invitrogen, Carlsbad, CA, USA) and mirVana™ miRNA Isolation Kit, without phenol (Invitrogen). In brief, 300 μL of serum was taken and centrifuged at 12,000× *g* for 10 min at 4 °C. Two hundred fifty microliters of supernatant was collected and homogenised with 750 μL of TRIzol™ LS Reagent. Two hundred microliters of chloroform was added to the homogenate, followed by a 3 min incubation at room temperature and subsequent centrifugation at 12,000× *g* for 15 min at 4 °C. Then, the upper aqueous layer was collected, followed by the addition of 650 μL of 100% ethanol. The mixture was then added to the filter cartilage provided in the mirVana™ miRNA Isolation Kit and was centrifuged at 10,000× *g* for 15 s. After washing once with 700 μL of Wash Solution 1 and twice with 500 μL of Wash Solution 2/3, the total RNA on the filter cartilage was eluted using 95 °C-preheated 30 μL RNase-free water.

### 4.3. Reverse Transcription (RT) and Quantitative Polymerase Chain Reaction (qPCR)

Total RNA was reversely transcribed using a combination of E. coli Poly(A) Polymerase (New England BioLabs, Ipswich, MA, USA) and PrimeScript RT reagent kit with gDNA eraser (Perfect Real Time) (Takara, Tokyo, Japan) to generate 20 μL of genomic DNA-free cDNA. To maximize the efficiency of miRNA detection, polyA tail was added to 2 μL of total RNA samples under 1X Reaction Buffer, 1 mM of ATP and 1.25 U of E. coli Poly(A) Polymerase, to generate 5 μL of the product after incubation at 37 °C for 30 min. Then, 1 μL of gDNA Eraser in 1X gDNA Eraser Buffer was added to the samples and was topped up to 10 μL with RNase-free water, followed by incubation at 42 °C for 2 min. Then, 1 μL of PrimeScript RT Enzyme Mix 1, 12.5 mM of the universal RT primer designed (5′-CAGGTCCAGTTTTTTTTTTTTTTTVN-3′) in 1X Buffer was used to generate 20 μL of cDNA after incubation at 37 °C for 15 min then 85 °C for 5 s.

To perform qPCR, TB Green Premix Ex Taq II (Tli RNaseH Plus) (Takara) was used together with miRNA-specific primers (Supplementary File S2), following the instructions of the manufacturer. In brief, 0.5 μL of gDNA-free cDNA and 0.4 μM of both forward and reverse primers were used per 10 μL reaction mix per well of a 96-well plate. Quantitative PCR was performed using ViiA7 Real-Time PCR System (ThermoFisher Scientific, Waltham, MA, USA) at 95 °C for 30 s, followed by 40 cycles at 95 °C for 5 s and 60 °C for 34 s.

### 4.4. Statistical Analysis

Descriptive statistics were summarized with mean and standard deviations. Intergroup comparisons were conducted by *t*-tests. All statistical analyses, including plotting of dot histograms, calculation of the area under the curve (AUC) of the receiver operating characteristic (ROC) curve for specific miRNA, and multiple linear regression, were conducted using SigmaPlot 10.0 (Systat Software Inc., San Jose, CA, USA). A *p*-value less than 0.05 (two-tailed) was considered statistically significant. 

### 4.5. Sample Size Calculation

Sample size calculation is performed by G*power 3.1 software [56]. Based on the results obtained from the serum miRNA score for NAFLD group and control group of this study, the calculated effect size was 0.5514. To achieve 80% power at a 5% significance level, the minimum number of samples in NAFLD and control group were 38 and 102, respectively.

## Figures and Tables

**Table 1:** Characteristics of colorectal polyp patients recruited.

	NAFLD Group (n = 38)	Control Group (n = 103)	*p*-Value
Male	22 (57.9%)	57 (55.3%)	
Female	16 (42.1%)	46 (44.7%)	0.936
Age	62.2 ± 8.50	64.9 ± 9.96	0.138
Type of polyp			
Hyperplastic	6	11	
Tubular adenoma	25	81	
Others	3	5	
Mixed types	4	6	0.470
Complications:			
Diabetes only	8	13	
Obesity/overweight only	5	14	
Diabetes and obesity/overweight	11	9	0.366

**Table 2:** Characteristics of colorectal polyp patients recruited in the validation cohort.

	Probable NAFLD Group (n = 10)	Control Group (n = 12)	*p*-Value
Male	7	9	
Female	3	3	1.000
Age	61.3 ± 5.95	63.8 ± 9.31	0.481
Type of polyp			
Hyperplastic	2	2	
Tubular adenoma	6	8	
Mixed types	2	2	0.949

## Data Availability

The data presented in this study are available on request from the corresponding author. The data are not publicly available due to privacy.

## Supplementary Materials

The following supporting information can be downloaded at: https://www.mdpi.com/article/10.3390/ijms24109084/s1.
